# Screening for metabolic dysfunction-associated steatotic liver disease in type 2 diabetes patients using the fibrosis-4 score in medina-retrospective study

**DOI:** 10.7717/peerj.21785

**Published:** 2026-10-21

**Authors:** Mohammed Abdullah Almohammadi, Ziyad Esam Alsadi, Ahmad Basheer Alenezi, Faisal Mahmoud Ahmed, Badr Mahmoud Ahmed, Radwan Mohamed Mulla, Nawaf Mohammed Alrehaili, Yosef Abdullah Alsaedi, Ahmed Saud Alhuwayfi, Amal Mohammed Surrati

**Affiliations:** 1 College of Medicine, Taibah University, Al Madinah, Saudi Arabia; 2 King Salman Bin Abdulaziz Medical City, Diabetic Center, MOH, Diabetes Center at KSAMC, Madianh, Saudi Arabia; 3 Family, Community and Medical Education Department, College of Medicine, Taibah University, Al Madinah, Saudi Arabia

**Keywords:** MASLD screening in type 2 diabetes using FIB-4, Screening, Type2 DM, FIB-4 score, Liver function

## Abstract

**Background:**

Type 2 diabetes mellitus (T2DM) is a global health problem closely linked to metabolic dysfunction-associated steatotic liver disease (MASLD) and advanced fibrosis. The fibrosis-4 (FIB-4) index is a standard noninvasive tool for estimating fibrosis risk. This study aimed to describe the demographics, metabolic parameters, comorbidities, and T2DM duration of patients in Medina and to screen for MASLD using the FIB-4 score.

**Methods:**

At King Salman Diabetic Center, Medina, Saudi Arabia, a cross-sectional study was conducted among 280 T2DM patients aged >35 years. Data were gathered from electronic medical records. FIB-4 levels were calculated and categorized as low (<1.3), intermediate (1.3–2.67), or high (>2.67) risk. Statistical analysis was performed with IBM SPSS Statistics 30, with *p* < 0.05 considered significant.

**Results:**

A total of 87.9%of the 280 participants had low-risk FIB-4 scores, 11.4% had intermediate risk scores, and 0.7% had high risk scores. Individuals with FIB-4 ≥ 1.3 were predominantly male (64.7% *vs*. 43.5%, *p* = 0.0271) and older (median age: 56 *vs*. 52 years, *p* = 0.009). The risk groups were not significantly different in terms of body mass index (BMI) and lipid profiles, glycemic control, hypertension, or diabetes duration.

**Conclusion:**

A significant proportion of T2DM patients had intermediate- or high-risk FIB-4 scores. These findings endorse the need for screening for liver fibrosis in T2DM patients, particularly those at increased risk. Early detection and appropriate management are essential to prevent progression to advanced liver disease.

## Introduction

Diabetes mellitus (DM) is among the top widespread chronic diseases globally, with the International Diabetes Federation (IDF) statistic as 537 million aged 20–79 years are affected (10.5% prevalence). In Saudi Arabia, 17.7% of adults were estimated to have diabetes by 2021, contributing to significant morbidity, mortality, complications, lifestyle changes, delayed diagnoses, and increased healthcare costs (International Diabetes Federation, 2021; Lovic et al., 2020; Orasanu & Plutzky, 2009).

Metabolic Dysfunction-Associated Steatotic Liver Disease (MASLD) Includes a wide range of related conditions, from simple hepatic steatosis with or without mild inflammation to metabolic Dysfunction-Associated Steatohepatitis (MASH) and even cirrhosis (European Association for the Study of the Liver, European Association for the Study of Diabetes, European Association for the Study of Obesity, 2016; Younossi et al., 2019).

Insulin resistance, a core feature of T2DM, contributes to MASLD development by promoting hepatic lipid accumulation and de novo lipogenesis (Smith et al., 2020; Valenti et al., 2016). Oxidative stress and inflammatory responses further contribute to diabetes-associated liver injury and disease progression (Mohamed et al., 2016).

T2DM is a major risk factor for developing MASH and accelerating liver disease progression, leading to worse outcomes (Younossi et al., 2019). Among individuals with T2DM, up to one-third (15–30%) may have clinically significant fibrosis, resulting in more than twice the risk of cirrhosis-related complications and liver disease mortality (Anstee, Targher & Day, 2013). Importantly, the extent of fibrosis is the strongest predictor of liver-related death and overall death (Ekstedt et al., 2015). Although many MASLD patients do not progress to cirrhosis, those with T2DM tend to progress more rapidly, with an elevated risk of MASH and liver-related mortality (Younossi et al., 2019).

MASLD is associated not only with hepatic complications but also with increased risks of cardiovascular disease, chronic kidney disease, and diabetic microvascular complications such as nephropathy, retinopathy, and neuropathy (Ziegler et al., 2018). The American Diabetes Association and the European Associations for the Study of the Liver, Diabetes, and Obesity recommended screening for advanced fibrosis in all patients with prediabetes or DM *via* noninvasive serum biomarkers for first-line risk stratification, identifying MASLD patients at low risk of advanced fibrosis/cirrhosis (European Association for the Study of the Liver, European Association for the Study of Diabetes, European Association for the Study of Obesity, 2016; Valenti et al., 2016).

The fibrosis-4 (FIB-4) index is a well-established, noninvasive marker for assessing liver fibrosis severity and is calculated on the basis of age, platelet count, aspartate aminotransferase (AST), and alanine aminotransferase (ALT) levels (Shah et al., 2009). Scores are classified as low (<1.3), intermediate (1.3–2.67), or high (>2.67) risk. Studies have shown that the FIB-4 score effectively predicts liver-related events and can be used to assess fibrosis severity, monitor disease progression, and guide clinical management (Ekstedt et al., 2015).

To our knowledge, this is the first study to assess FIB-4-based MASLD screening among T2DM patients in Medina, Saudi Arabia, thereby providing context-specific evidence for local healthcare planning. Therefore, this study aimed to evaluate the prevalence of elevated FIB-4 scores among patients with T2DM in Medina, Saudi Arabia, as an initial screening approach for MASLD. Understanding the burden of liver fibrosis risk in this high-risk population is essential for guiding early detection strategies, optimizing patient management, and informing local healthcare policies.

## Methods

### Study design and setting

This retrospective cross-sectional study was conducted at the King Salman Diabetic Center (KSDC) in Almadinah Almunawwarah, Saudi Arabia. Data were collected from outpatient electronic medical records between January and August 2024. Eligible participants were identified on the basis of the inclusion and exclusion criteria. Laboratory and clinical data were collected from the most recent outpatient visit within the study period. The extracted data included demographics, sex, anthropometric measurements, laboratory results (complete blood count, liver function tests, metabolic parameters), and documented comorbidities such as cardiovascular disease, renal disease, hypertension, and obesity.

### Study population

Based on an estimated adult population of 684,743 in Almadinah Almunawwarah (2022 Census), a 17.7% diabetes incidence, and a 95% CI, the minimum required sample size was 224 (Raosoft Inc., 2026).

The inclusion and exclusion criteria were as follows:

Inclusions were those aged >35 years, known T2DM according to The American Diabetes Association (ADA) criteria, and availability of blood test results.

The exclusion criteria included pregnancy; history of cardiovascular disease; liver fibrosis, hepatitis, thrombocytopenia, or chronic kidney disease; and missing key variables for FIB-4 calculations.

These factors were excluded from the analysis of hepatic fibrosis risk independent of preexisting conditions.

### Cutoff values

Body mass index (BMI) was classified according to The World Health Organization (WHO) guidelines: underweight (<18.5 kg/m^2^), normal (18.5–24.9), overweight (25.0–29.9), obese class I (30.0–34.9), class II (35.0–39.9), and class III (>40).

Normal laboratory reference ranges according to the KSDC laboratory are as follows: AST 0–41 U/L, ALT 0–35 U/L, and platelets 150–450 × 10^9^/L. The lipid profiles were as follows: total cholesterol 0–5.18 mmol/L; high-density lipoprotein (HDL) 0–2.59 mmol/L; low-density lipoprotein (LDL) ideally up to 3.37 mmol/L; and triglycerides 0–1.7 mmol/L.

FIB-4 was calculated as (Age [years] × AST [U/L])/(Platelet count [10^9^/L] × √ALT [U/L]) (MDCalc, 2025). Glycemic control is defined according to the ADA Standards of Care 2025 as an HbA1c less than 7% and fasting blood sugar (FBS) between 4.4 and 7.2 mmol/L (American Diabetes Association Professional Practice Committee, 2025).

### Statistical analysis plan

Analyses were performed *via* IBM SPSS Statistics version 30 (IBM Corp., Armonk, NY, USA). Continuous variables are reported as the mean ± standard deviation if normally distributed or as the median and interquartile range (IQR) if skewed. Categorical variables are summarized as frequencies and percentages. Comparisons between groups used independent t tests for normally distributed data, Kruskal–Wallis H tests for nonnormal data, and chi-square tests or exact tests for categorical variables. A *p* value of <0.05 was considered to indicate statistical significance.

No imputation was performed for missing data; cases with missing key variables were excluded from the analysis.

### Ethics statement

This study was conducted in accordance with the principles of the Declaration of Helsinki. The protocol was approved by the Institutional Review Board of King Salman bin Abdulaziz Medical City on December 24, 2023 (IRB number: 23-066). The need for informed consent was waived because of the retrospective nature of the study. All the data were anonymized before analysis to maintain participant confidentiality. Access to electronic medical records was restricted to authorized research team members, and the data was stored securely in password-protected files in accordance with institutional data protection policies.

## Results

### T2DM patient characteristics

A total of 280 patients were included. Based on FIB-4 scores, 246 (87.9%) patients were classified as low risk, 32 (11.4%) as intermediate risk, and 2 (0.7%) as high risk. This study included 129 males (46.1%) and 151 females (53.9%), with a median age of 53 years (IQR: 13). Saudi nationals accounted for 92.5% of the participants. The median BMI was 31.05 kg/m^2^.

The histogram shows a right-skewed distribution of FIB-4 scores, with clustering between 0.5 and 1.5, and the highest frequency is approximately 1.0 (Fig. 1).

**Figure 1:**
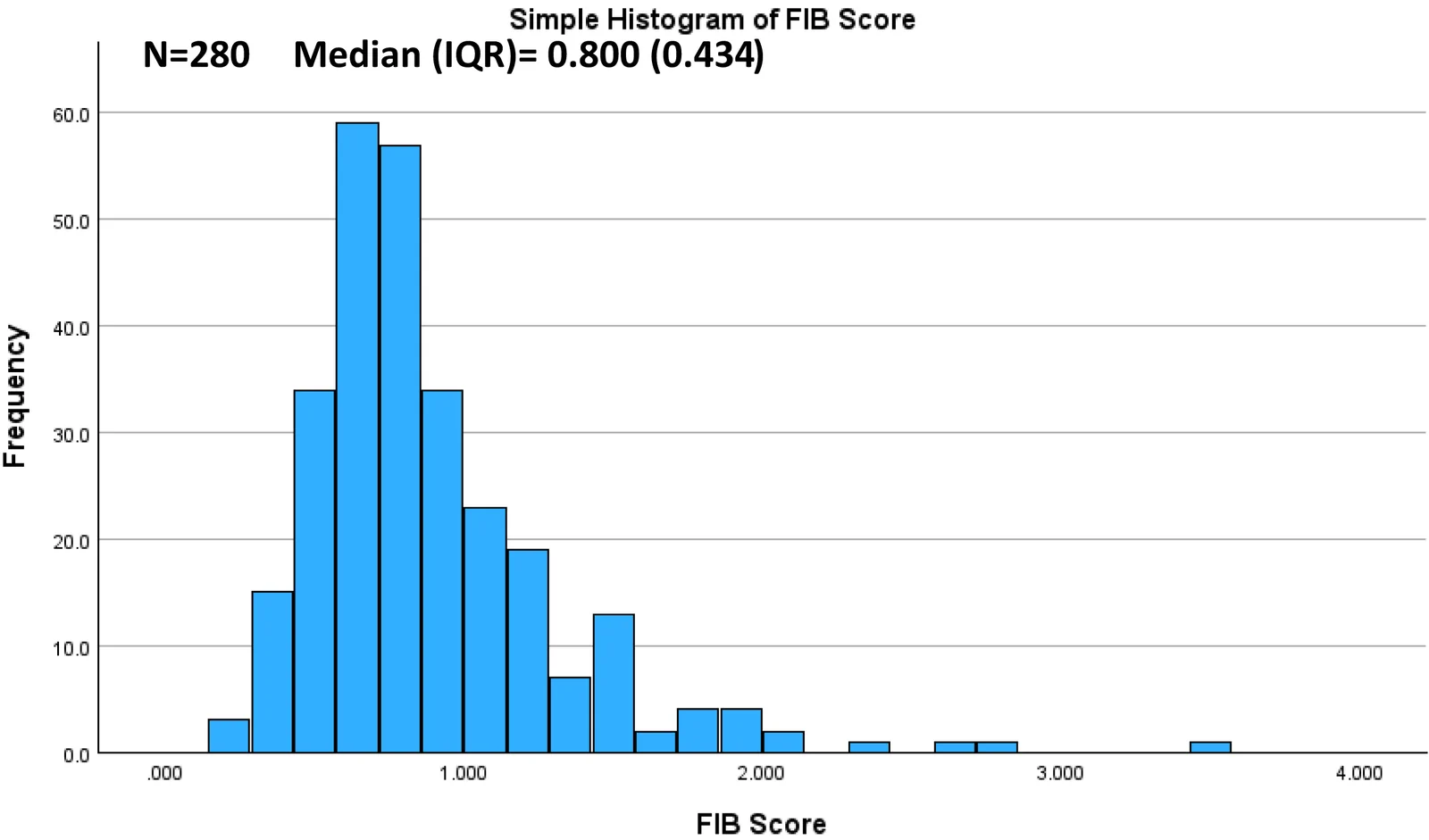
Histogram of FIB-score. Distribution of FIB-4 scores among 280 patients with type 2 diabetes mellitus (T2DM). The histogram demonstrates a right-skewed distribution, with most scores clustered between 0.5 and 1.5 and the highest frequency observed at approximately 1.0. The median (IQR) FIB-4 score was 0.800 (0.434). IQR = interquartile range.

With respect to metabolic measures, 68.6% of the participants had total cholesterol ≤5.18 mmol/L, whereas HDL was <1.04 mmol/L in 23.2% of the participants. LDL was ≤2.59 mmol/L in 50.4% of the patients, and triglycerides were >1.7 mmol/L in 47.9%. Glycemic control was not achieved (HbA1c ≥ 7%) in 77.9% of patients. Hypertension was present in 41.8% of the patients. The median duration since T2DM diagnosis was 9 years. The baseline characteristics and FIB-4 risk stratification are summarized in Table 1.

**Table 1:** Baseline characteristics and risk stratification of the study participants based on FIB score categories.

Characteristic	Overall	Low Risk FIB score < 1.3	Intermediate Risk FIB score 1.3–2.67	High Risk FIB score > 2.67
	*N* = 280	*N* = 246 (87.9%)	*N* = 32 (11.4%)	*N* = 2 (0.7%)
Gender				
Male	129 (46.1%)	107 (38.2%)	20 (7.1%)	2 (0.7%)
Female	151 (53.9%)	139 (49.6%)	12 (4.4%)	0
Age in years	53 (13)			
Nationality				
Saudi	259 (92.5%)	229 (81.8%)	28 (10%)	2 (0.7%)
Non-Saudi	21 (7.5%)	17 (6.1%)	4 (1.4%)	0
BMI (kg/m^2^)	31.05 (8.75)			
Normal weight	36 (12.9%)	30 (10.7%)	5 (1.8%)	1 (0.35%)
Preobesity	88 (31.4%)	77 (27.5%)	11 (3.9%)	0
Obesity class I BMI = 30.0–34.9	81 (28.9%)	70 (25%)	10 (3.6%)	1 (0.35%)
Obesity class II BMI = 35.0–39.9	52 (18.6%)	47 (16.8%)	5 (1.8%)	0
Obesity class III BMI > 40	23 (8.25%)	22 (7.9%)	1 (0.35%)	0
Liver parameters				
AST (U/L)	19.5 (8)			
AST ≤ 41	270 (96.4%)	244 (87.1%)	26 (9.3%)	0
AST > 41	10 (3.6%)	2 (0.7%)	6 (2.1%)	2 (0.7%)
ALT (U/L)	20 (12)			
ALT ≤ 35	246 (87.9%)	226 (80.7%)	20 (7.1%)	0
ALT > 35	34 (12.1%)	20 (7.1%)	12 (4.3%)	2 (0.7%)
Platelets (10^9^/L)	280.5 (101.8)			
<150	3 (1.1%)	0	1 (0.35%)	2 (0.7%)
150–450	267 (95.4%)	236 (84.3%)	31 (11.1%)	0
>450	10 (3.5%)	10 (3.5%)	0	0
Metabolic parameters				
Total cholesterol (mmol/L)	4.75 (1.63)			
≤5.18	192 (68.6%)	170 (60.7%)	21 (7.5%)	1 (0.05%)
>5.18	88 (31.4%)	76 (27.1%)	11 (3.9%)	1 (0.35%)
HDL (mmol/L)	1.24 (0.3)			
<1.04	65 (23.2%)	54 (19.3%)	10 (3.5%)	1 (0.35%)
1.04–1.55	188 (67.1%)	166 (59.3%)	21 (7.5%)	1 (0.35%)
>1.55	27 (9.65%)	26 (9.3%)	1 (0.35%)	0
LDL (mmol/L)	2.52 (1.2)			
≤2.59	141 (50.4%)	122 (43.6%)	18 (6.4%)	1 (0.35%)
>2.59	139 (49.6%)	124 (44.3%)	14 (5%)	1 (0.35%)
Triglycerides (mmol/L)	1.69 (0.89)			
≤1.7	146 (52.1%)	129 (46.1%)	17 (6%)	0
>1.7	134 (47.9%)	117 (41.8%)	15 (5.4%)	2 (0.7%)
Status of glycemic control				
A1C (%)	7.7 (1.9)			
Fasting blood sugar (mmol/L)	7.5 (3.65)			
Not controlled	218 (77.9%)	191 (68.2%)	25 (8.9%)	2 (0.7%)
Controlled	62 (22.1%)	55 (19.6%)	7 (2.5%)	0
Presence of hypertension				
No	163 (58.2%)	141 (50.4%)	21 (7.5%)	1 (0.35%)
Yes	117 (41.8%)	105 (37.5%)	11 (3.9%)	1 (0.35%)
Years since diagnosis with T2DM	9 (8)			

**Note:**

*N* (%); Median (IQR).

### FIB-4 score and demographics

Among the 280 patients, 34 (12.1%) had FIB-4 scores ≥1.3. The proportion of males was greater in the ≥1.3 group than in the <1.3 group (64.7% *vs*. 43.5%, *p* = 0.027). The median age was greater in the ≥1.3 years group (56 years) than in the <1.3 years group (52 years) (*p* = 0.009) (Fig. 2).

**Figure 2:**
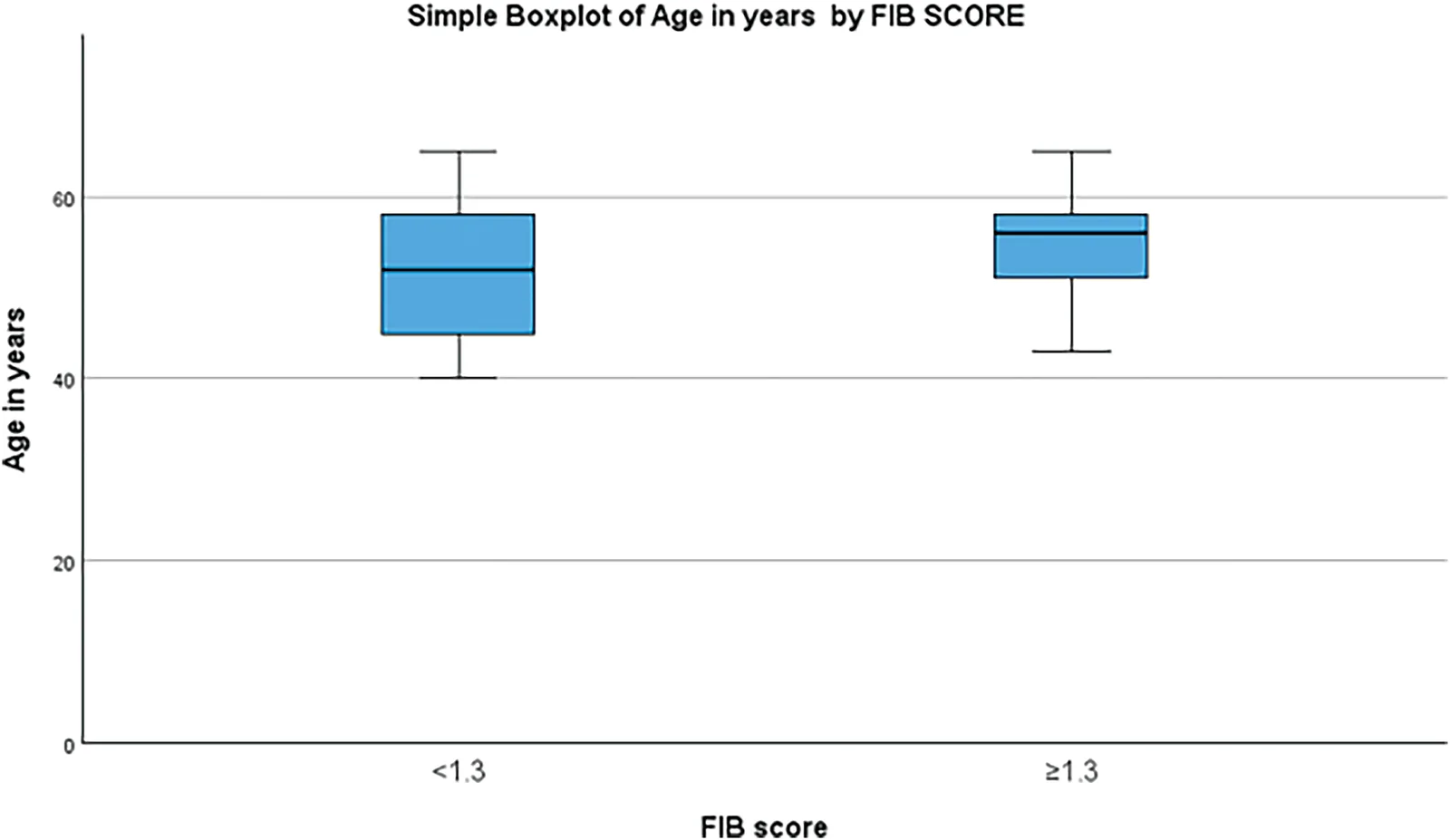
Simple boxplot of age in years by FIB score. Comparison of age between patients with FIB-4 scores ≥1.3 (*N* = 34) and those with scores <1.3 (*N* = 246). The median (IQR) age was 56 (8) years in the ≥1.3 group and 52 (13) years in the <1.3 group. The difference was statistically significant (*P < 0.05; Mann-Whitney U test). IQR = interquartile range.

### FIB-4 score and liver parameters

Elevated AST (>41 U/L) was more common in patients with FIB-4 ≥1.3 (23.5%) than in those with FIB-4 <1.3 (0.8%) (*p* < 0.001). Elevated ALT levels (>35 U/L) were also more common in the ≥1.3 group (41.2% *vs*. 8.1%, *p* < 0.001). Thrombocytopenia (platelets < 150 × 10^9^/L) occurred only in the ≥1.3 group (8.8%, *p* < 0.001).

### FIB-4 score and metabolic parameters

BMI showed no statistically significant difference between the two groups. No significant differences were observed in total cholesterol, HDL, LDL, triglyceride, HbA1c, fasting blood glucose, hypertension incidence, or years since T2DM diagnosis. The comparative characteristics of the FIB-4 categories are shown in Table 2.

**Table 2:** Comparison of demographic, clinical, and metabolic characteristics by FIB score category.

Characteristic	Overall	FIB score < 1.3	FIB score ≥ 1.3	*p* value
	*N* = 280	*N* = 246 (87.85%)	*N* = 34 (12.15%)	
Gender				0.027^1^
Male	129 (46.1%)	107 (43.5%)	22 (64.7%)	
Female	151 (53.9%)	139 (56.5%)	12 (35.3%)	
Nationality				0.223^1^
Saudi	259 (92.5%)	229 (93.1%)	30 (88.2%)	
Non-Saudi	21 (7.5%)	17 (6.9%)	4 (11.8%)	
Age in years	53 (13)	52 (13)	56 (8)	0.009^2^
BMI (kg/m^2^)	31.05 (8.75)			
Normal weight	36 (12.9%)	30 (12.2%)	6 (17.6%)	0.199^1^
Preobesity	88 (31.4%)	77 (31.3%)	11 (32.4%)	
Obesity class I	81 (28.9%)	70 (28.5%)	11 (32.4%)	
Obesity class II	52 (18.6%)	47 (19.1%)	5 (14.7%)	
Obesity class III	23 (8.2%)	22 (8.9%)	1 (2.9%)	
Liver parameters				
AST (U/L)	19.5 (8)			>0.001^1^
AST ≤ 41	270 (96.4%)	244 (99.2%)	26 (76.5%)	
AST > 41	10 (3.6%)	2 (0.8%)	8 (23.5%)	
ALT (U/L)	20 (12)			>0.001^1^
AST ≤ 35	246 (87.9%)	226 (91.9%)	20 (58.8%)	
AST > 35	34 (12.1%)	20 (8.1%)	14 (41.2%)	
Platelets (10^9^/L)	280.5 (101.8)			>0.001^1^
<150	3 (1.1%)	0	3 (8.8%)	
150–450	267 (95.4%)	236 (95.9%)	31 (91.2%)	
>450	10 (3.5%)	10 (4.1%)	0	
Metabolic parameters				
Total cholesterol (mmol/L)	4.75 (1.63)	4.75 (1.54)	4.57 (2.48)	0.500^2^
≤5.18	192 (68.6%)	170 (69.1%)	22 (64.7%)	
>5.18	88 (31.4%)	76 (30.9%)	12 (35.3%)	
HDL (mmol/L)	1.24 (0.3)	1.24 (0.29)	1.26 (0.36)	0.681^2^
<1.04	65 (23.2%)	54 (22.0%)	11 (32.4%)	
1.04–1.55	188 (67.1%)	166 (67.5%)	22 (64.7%)	
>1.55	27 (9.65%)	26 (10.6%)	1 (2.9%)	
LDL (mmol/L)	2.52 (1.2)	2.60 (1.2)	2.41 (1.94)	0.908^2^
≤2.59	141 (50.4%)	122 (49.6%)	19 (55.9%)	
>2.59	139 (49.6%)	124 (50.4%)	15 (44.1%)	
Triglycerides (mmol/L)	1.69 (0.89)	1.66 (0.85)	1.71 (1.13)	0.652^2^
≤1.7	146 (52.1%)	129 (52.4%)	17 (50.0%)	
>1.7	134 (47.9%)	117 (47.6%)	17 (50.0%)	
Status of glycemic control				
A1C (%)	7.7 (1.90)	7.7 (1.92)	7.7 (1.72)	0.407^2^
Fasting blood sugar (mmol/L)	7.50 (3.65)	7.53 (3.72)	7.54 (2.96)	0.625^2^
Not controlled	218 (77.9%)	191 (77.6%)	27 (79.4%)	0.833^1^
Controlled	62 (22.1%)	55 (22.4%)	7 (20.6%)	
Presence of hypertension				0.462^1^
No	163 (58.2%)	141 (57.3%)	22 (64.7%)	
Yes	117 (41.8%)	105 (42.7%)	12 (35.3%)	
Years since diagnosis with T2DM	9 (8)	9 (9)	10 (7)	0.662^1^

**Notes:**

*N* (%); Median (IQR).

1 Pearson’s Chi-squared test/Fisher’s exact test.

2 Mann‒Whitney test.

## Discussion

This study evaluated 280 patients with T2DM in Medina, Saudi Arabia, to describe their demographic, metabolic, and comorbidity profiles and to identify risk factors for elevated FIB-4 scores in the context of MASLD screening. To our knowledge, this is among the few studies in Saudi Arabia to specifically report the pattern of FIB-4 score variation among individuals with type 2 diabetes mellitus, providing valuable baseline data for regional screening strategies.

The majority of patients (87.9%) were classified as low risk on the basis of the FIB-4 index, with 11.4% at intermediate risk and only 0.7% at high risk. These proportions are comparable to those reported in a retrospective cohort of 12,589 T2DM patients, who reported elevated hepatic fibrosis risks of 14.1% according to the BMI, AST/ALT ratio, and diabetes (BARD) score, 8.7% according to the MASLD fibrosis score, 5.4% according to the FIB-4 index, and 3.4% according to the AST-to-Platelet Ratio Index (APRI) score (Naskar et al., 2024). Minor differences may reflect variations in sample size, population demographics, and the scoring systems used. The FIB-4 index, which incorporates AST, ALT, and platelet count, remains a widely used, cost-effective, noninvasive marker for liver fibrosis in T2DM patients (Kim et al., 2024).

Male sex was strongly associated with elevated FIB-4 scores (≥1.3), with males comprising 64.7% of this group. This finding aligns with previous research showing increased risks of advanced hepatic fibrosis among male T2DM patients (Kaya et al., 2020). Although the diagnostic performance of FIB-4 is generally comparable across genders, age-related influences may lead to overestimation of risk in older adults, prompting recommendations for age-adjusted cutoffs (Chen, 2021; Mueller, 2020).

Because age is a key variable in the FIB-4 calculation, the finding that patients with elevated FIB-4 scores were older was anticipated; the median age was 56 years in the elevated-risk group compared with 52 years in the low-risk group.

This finding is consistent with large-scale studies linking older age to higher FIB-4 scores and advanced fibrosis risk, especially in patients with T2DM and obesity (Anstee et al., 2023). Age effects on FIB-4 interpretation highlight the importance of considering adjusted cutoffs to improve specificity in older populations (Chen, 2021).

Previous studies have shown that liver enzyme elevation is associated with higher BMI and poorer metabolic control in T2DM patients (Forlani et al., 2008). Our findings of BMI showed no significant statistical variation between the groups. A slightly greater prevalence of obesity was observed among those with elevated scores. These findings support the role of weight management in mitigating liver-related complications.

No significant differences in cholesterol, HDL, LDL, or triglyceride levels were found between the FIB-4 groups, despite nearly half of the participants having hypertriglyceridemia and one-quarter having low HDL. These findings may reflect reasonable lipid control or insufficient adjustment for other comorbidities. Nonetheless, dyslipidemia remains a common cardiovascular risk factor in T2DM patients, and previous studies have linked certain lipid patterns to increased fibrosis risk (Forlani et al., 2008; Zhang et al., 2023).

Hypertension was not significantly associated with elevated FIB-4 scores in our study. This aligns with the results reported in a previous Mexican study that reported that hypertension was not an independent risk factor of fibrosis in T2DM patients (Martínez-Sánchez et al., 2025). However, other studies, such as that of Sato et al. (2022), reported a positive relationship between hypertension incidence and the FIB-4 score. Differences in findings may reflect population characteristics, FIB-4 cutoffs, or statistical adjustments.

The duration of T2DM did not differ significantly between risk groups, in contrast to several studies linking longer disease duration to fibrosis progression (Kawata et al., 2020). Chronic hyperglycemia, insulin resistance, and systemic inflammation are proposed mechanisms for this association. While some studies support this link (Anstee et al., 2023), other studies report inconsistent correlations as multiple interacting factors play a role (Cernea, 2024).

FIB-4 is considered a useful first-line, noninvasive screening tool for advanced fibrosis in patients with T2DM, regardless of duration of diabetes, particularly in routine clinical practice (Gracen et al., 2022).

In our sample, 77.9% of patients did not achieve glycemic control, and 41.8% were hypertensive—both with established cardiovascular and microvascular risk factors. Although this study did not include other comorbidities, such as cardiovascular or renal disease, previous evidence indicates that elevated FIB-4 scores predict higher mortality and progression to end-stage kidney disease in T2DM patients (Forlani et al., 2008), as well as greater cardiovascular disease burden (Zhao et al., 2024; Méndez-Sánchez et al., 2020).

In summary, 12.1% of patients in this cohort had FIB-4 scores ≥1.3, identifying a subgroup that may warrant further fibrosis risk assessment. This group was predominantly older and male. Variations in liver enzymes and platelet counts were observed in parallel with higher FIB-4 scores, reflecting the components of the index rather than independent clinical associations. No significant differences were identified in BMI, lipid profiles, glycemic control, hypertension prevalence, or duration of T2DM. These results align with earlier reports of similar HbA1c distributions across FIB-4 categories (Chun et al., 2021).

### Strengths and limitations

This study provides the first detailed description of the FIB-4 score distribution and associated risk factors in T2DM patients from Medina, contributing to regional MASLD risk assessment. The strengths of this study include an adequate sample size exceeding power calculations, robust statistical analysis, and a clear presentation of the results. Limitations include the retrospective, single-center design, reliance on medical records (limiting available variables such as cardiovascular history), and the absence of a gold-standard diagnostic confirmatory test, such as imaging or biopsy for fibrosis staging, as validation for the FIB-4 score.

The cross-sectional design also precludes causal inference.

## Conclusion and recommendations

Higher FIB-4 scores (≥1.3) in patients with T2DM from Medina were predominantly seen in older males and were associated with expected variations in transaminase levels and platelet counts, consistent with the structure of the FIB-4 index.

Although BMI, lipid profiles, glycemic control, and hypertension did not differ significantly between the groups, these results support the routine use of FIB-4 as a first-line, noninvasive screening tool in T2DM care. Early identification of at-risk patients may allow timely interventions to prevent MASLD progression.

FIB-4 serves as a first-line triage tool, and that patients with elevated scores should undergo further definitive assessment, such as transient elastography or specialist referral, to confirm liver fibrosis and guide management.

Future longitudinal, multicenter studies in Saudi Arabia are warranted to validate these findings, explore additional risk factors, and assess the impact of lifestyle and pharmacological interventions.

## Supplemental Information

10.7717/peerj.21785/supp-1 Supplemental Information 1 Raw data.

10.7717/peerj.21785/supp-2 Supplemental Information 2 Codebook.

## Additional Information and Declarations

### Data Availability

The following information was supplied regarding data availability:

The raw measurements are available in the Supplemental Files.
