# Supplementary material for: Screening for metabolic dysfunction-associated steatotic liver disease in type 2 diabetes patients using the fibrosis-4 score in medina-retrospective study
Source: PeerJ. 2026 Oct 21;14:e21785. doi: 10.7717/peerj.21785 (PMC13695418; doi:10.7717/peerj.21785)
Supplement: Supplementary file 1 [file peerj-14-21785-s002.pdf]

Codebook	
**Variable / Number**	**Respective factor**
**Nationality**	
**0**	Non-Saudi
**1**	Saudi
**Gender**	
**1**	Male
**2**	Female
**HTN**	
**0**	Didn't diagnose with HTN
**1**	Diagnosed with HTN
**Medication**	
**0**	Non-insulin alone
**1**	Insulin alone
**2**	Both insulin and non-insulin
**Biguanides**	
**0**	Does not take the medication
**1**	Take the medication
**SGLT2Inhibitor**	
**0**	Does not take the medication
**1**	Take the medication
**Sulfonylureas**	
**0**	Does not take the medication
**1**	Take the medication
**DPP4Inhibitor**	
**0**	Does not take the medication
**1**	Take the medication
**GLP1**	
**0**	Does not take the medication
**1**	Take the medication
